# *How Self-Determined Are Reproductive Decisions*? Sociological Aspects of Pregnancy, Birth, and Breastfeeding: Implications for Midwifery Practice—A Narrative Review

**DOI:** 10.3390/healthcare13131540

**Published:** 2025-06-27

**Authors:** Joachim Graf, Konstanze Weinert, Harald Abele, Angela Kranz

**Affiliations:** 1 Institute for Health Science, Department of Midwifery Science, University Hospital Tuebingen, 72076 Tuebingen, Germany; konstanze.weinert@med.uni-tuebingen.de (K.W.); harald.abele@med.uni-tuebingen.de (H.A.); angela.kranz@med.uni-tuebingen.de (A.K.); 2 Department of Women’s Health, University Hospital Tuebingen, 72076 Tuebingen, Germany

**Keywords:** health sociology, social construction of pregnancy, birth and breastfeeding, midwives, self-determination of reproductive decisions

## Abstract

Pregnancy and birth are biological processes shaped by social factors, requiring sociological approaches to explain reproductive behaviour. This narrative review outlines the importance of health sociology against the background that health and illness behaviour is influenced by the social environment. The aim of this paper is to summarize the current state of research on the influence of social systems and social milieu behaviour on reproduction, pregnancy, and childbirth in order to make it easier for midwives and doctors to take these factors into account in their everyday clinical and outpatient work. First, the paper lays out the basics of how health and illness are socially constructed, looking at it from both a structural and action-oriented perspective. It then goes on to explain what this means for pregnancy and childbirth as social processes, how women’s health is related to the social construction of gender roles, that breastfeeding is also a social process, and what conclusions can be drawn for the work of midwives. Pregnancy and birth are social processes based on norms and role attributions: “Decisions” regarding one’s own reproductivity are usually only “self-determined” to a limited extent and tend to occur in the context of social norms and milieu-specific role expectations. The promotion of women’s health depends on how milieu-specific norms and logics of action are understood. For all the professions involved in obstetrics, this results in the need for a critical examination of the sociological aspects of health. This implies the necessity for all obstetric professions to critically examine aspects of the sociology of health in order to provide women and their families with appropriate, evidence-based and client-centred care in the context of pregnancy, birth and the postpartum period, against the background of constant social change.

## 1. Introduction

Pregnancy and birth are physiological and biological processes which are also influenced by social factors, which is why sociological approaches are needed to explain reproductive behaviour. Sociology emphasizes the influence of norms, beliefs and behaviour patterns on collective and individual action and recognizes that these are constantly changing; being repeatedly re-evaluated, re-interpreted and re-adapted; and are interpreted and reproduced differently depending on social milieu and culture. Pregnancy and birth are social events which take place in surrounding social subsystems (at micro, meso and macro levels) and are therefore centrally influenced by existing norms and socially constructed ritualized behaviours [1]. This means that the willingness to follow health-promoting recommendations from health professionals (such as midwives and gynaecologists) also depends on the social systems surrounding pregnant women. It can therefore be assumed that there is an increased risk of inadequate, excessive, or insufficient care if professionals do not take social circumstances sufficiently into account [1,2,3]. Although the influence of social factors on health and reproductive behaviour has been well researched from a sociological perspective, there is a lack of overview articles that summarize relevant aspects from a health science perspective. 

The aim of this paper is to summarize the current state of research on the influence of social systems and social milieu behaviour on reproduction, pregnancy and childbirth in order to make it easier for midwives and doctors to take these factors into account in their everyday clinical and outpatient work. The present review outlines the importance of health sociology against the background that health and illness behaviour is influenced by the social environment. On the basis of specific examples, the article shows how these factors result in a social construction of pregnancy, birth and lactation and discusses relevant implications for midwifery, since midwives are part of the gender and family sociology discourse.

## 2. Methods

This paper was designed as a narrative review. The aim was not to evaluate the evidence provided in existing papers or to quantify or cluster the content of existing publications but rather to summarize the current state of research in terms of content. The sociological perspective was to be “translated” into a health science perspective in order to make the findings useful for all professionals working in obstetrics. The literature search was correspondingly selective and unsystematic. Research was conducted in PubMed and Google Scholar. Because health sociology publications often serve to develop theory and the number of scientific interventions is lower than in many areas of medical research reviews must cover longer periods of time. Accordingly, this paper took into account studies published since the 1980s in either scientific journals or specialist books. All study designs were included, provided they were relevant to the research question focused on here. We included studies in English and German. The following search terms (some combined using Boolean operators) were used for the research: “sociology of pregnancy”, “sociology of birth”, “health sociology”, “social determinants”, “breastfeeding as a social process”, “structural functionalism in the context of reproduction, pregnancy, birth, and breastfeeding”, “action theories in the context of reproduction, pregnancy, birth, and breastfeeding”, “sexual dispositif”, “gender role”, “gender sociology”, “gender role and reproduction”, “sociology and midwifery”, “social construction/production of reproduction, pregnancy, birth, and breastfeeding”, “self-determined reproductive/sexuality and social norms”, “norms and reproduction”, “social expectations and reproduction”, “social milieu and reproduction, pregnancy, birth, and breastfeeding”. Separate research was conducted for each of the chapters. As part of this multi-stage process, 253 papers were initially identified and reviewed, and a total of 136 papers were considered.

## 3. Social Construction of Health and Illness

The sociology of health is fundamentally concerned with the analysis of social conditions of health and illness. The focus lies on the social conditions for health and illness at the level of the social and healthcare system (macro level), as well as on the perceptions of health and illness that are reproduced in the social subsystems in a milieu-specific way (meso level), and on the socioeconomic status of people and its influence on their health and illness (micro level) [2,3]. The following section will outline the fundamentals of health-related behaviours from both a structuralist and an action theory perspective. Structuralism and action theory are two different approaches in the social sciences that differ in their basic assumptions and focus with regard to the extent to which an individual’s actions are dependent on their social environment [1,2,3,4]. Structuralists emphasize that the thoughts and actions of individuals (including behaviours in the context of health and illness) are shaped by social systems (e.g., the healthcare system, the education system, but also the family or workplace) [1,2,3,4]. Action theory, on the other hand, focuses on individual actors and their motivations, goals and actions and would argue that the thoughts and actions of individuals depend, among other things, on individual goals based on a cost–benefit analysis, e.g., in the context of a desired social repositioning [1,2,3,4].

*Structuralist theories* postulate that health and illness, as well as how they are conceptualized, are influenced by supra-individual social structures and are thus modulated by society and its diverse subsystems at the micro-, meso- and macro-sociological levels. Structuralists would therefore look at the socially constructed demarcation between health and illness, analysing the conditions under which an individual is considered “sick” or “healthy” and how these constructions are shaped by social structures, cultural norms, and social power relations [4,5,6]. This is linked to discourses on how to deal with people who are classified as ill, for example, under what conditions a doctor issues a sick note and when specific interventions are carried out, but also what role expectations have to be fulfilled by people who are now acting as patients [4,6,7,8]. Talcott Parsons defined health as a requirement for the maintenance of society and illness accordingly as a disruption of the normal functioning of society [4,8]. The restoration of health (which presupposes that a defined disease status has been medically identified) is accordingly a social task to eliminate illness, understood as a social deviation from the “normal”, i.e., the healthy [7,8]. Society releases people in the role of patients from individual role obligations, for example, by means of sick leave [4,8]. At the same time, there is an expectation that sick people will want to recover. This means that they should seek expert help and follow medical advice [4,6,7,8]. The paternalistic relationship model underlying this role expectation has given way in the 21st century to participatory decision-making. However, concepts such as compliance and adherence ultimately also require the patient’s will to get well and their factual subordination to professional expertise, because the treatment pathway is determined within a narrow corridor of evidence-based medicine in negotiations between the patient and the medical experts [4,6,7,8,9,10,11]. The demarcation between health and illness varies from one culture to another and has constantly changed over time, as Michel Foucault, for example, has very explicitly shown using the development of psychiatry and its treatment of homosexuality [12]. Until the 1990s, homosexuality was still defined in the ICD-10 catalogue as a pathological deviation from sexuality defined as “normal” and accordingly as a clinical picture worthy of treatment [13,14].

*Action theory approaches* follow the basic assumption that “illness” and “health” are influenced by the actions of the actors from a micro sociological perspective [15,16,17]. This means that the health behaviour (e.g., in terms of the relevance attributed to prevention) and risk behaviour (e.g., the probability of smoking or recognizing smoking as a positive behaviour) of the individual are centrally dependent on the prevailing norms of the respective social environment in which the individual has experienced socialization. Furthermore, this means that aspects of health and illness are charged with (also milieu-specific) communicative symbols [15,16,17]. There is plenty of evidence confirming this point: life expectancy, probability of disease and morbidity are largely dependent on social situation and milieu affiliation [18,19,20]. Taking Germany as an example, there is a difference of almost 10 years in average life expectancy depending on income for both men and women: Women from a family in the lowest income group (<60% of net equivalent income, i.e., classified as at risk of poverty) can expect a mean life expectancy at birth of 76.6 years, while women from the highest income group (≥150% of net equivalent income) can expect 85.3 years [18,21]. Corresponding correlations have also been demonstrated for the USA [22]. High income is usually associated with a higher level of education and greater cultural and social capital and is significantly more common in socially advantaged milieus than in those with low socioeconomic status. There are therefore many correlations between social situation and health [23]. People with a low socioeconomic status are more often affected by multimorbidity [24] and chronic illnesses such as type 2 diabetes mellitus [25], coronary heart disease (CHD) [26], COPD [27] or depression [28] and rate their general health situation (subjective health) as worse than people with a low level of deprivation [29]. Affiliation to a particular milieu or stratum thus reproduces social inequality and has a significant influence on health, since it moderates all central areas of life that also influence health and health behaviour, e.g., nutrition, exercise, working and living conditions. This results in a social production of health and illness: *social production* means that (health) resources are distributed unequally. People differ in terms of their health resources (related to factors such as educational status, health literacy, living and working conditions) [7,21,30,31,32]. There is also a *social construction* of health, which refers to the fact that health-related behaviour is strongly influenced by an individual’s knowledge and ideas about health and disease, which in turn are influenced by the social systems in which the individual is embedded [7,21,30,31,32]. From a midwife’s perspective, it can be said that decisions and decision-making processes relating to health and illness are influenced by a wide range of social factors and are not the result of completely autonomous decision-making by individuals. This also applies to the likelihood of falling ill and coping with the consequences of illness.

## 4. Pregnancy and Birth as Social Processes

Social factors influence both health and illness in the context of pregnancy and birth. Pregnancy and midwifery are also determined by social structures as well as by the agency of individuals [1].

From a *structuralist perspective*, the focus is on the functionalization associated with the re-attribution of roles for the person who has now been identified as pregnant: Within the macro-system, it is defined which institutionalized rules of behaviour are imposed on pregnant women in the context of the moral obligation to become or remain healthy (since pregnancy and childbirth are not defined as illnesses in social law), for example, regarding preventive medical check-ups or maternity protection guidelines, which explicitly prescribe which professional activities are allowed under which conditions and up to which week of pregnancy [1,6,15,33]. Despite the trend towards “self-determined pregnancy” and “participatory decision-making”, there is also a social expectation that women will seek the advice of experts such as midwives and doctors [1,6,15,33]. “Self-determination” does not refer to the absence of social pressure (such an absence is sociologically impossible because reproduction is also a social activity that depends on norms) but merely to the autonomy granted within the respective social systems for decisions within the healthcare system. Autonomy depends, among other things, on the level of education, health literacy, cultural norms, milieu-specific role expectations, and institutional requirements of the healthcare system [1,6,15,33]. Macro sociological interest also centres on the demarcation of boundaries, e.g., when prenatal diagnostics reveal foetal anomalies that, in extreme cases, justify a late-term abortion (i.e., when, in a sociological sense, an inability to live is postulated) [34,35,36,37]. This demarcation was subject to constant change (due to continuous medical and technical progress), and it can be assumed that there will continue to be regular re-evaluations and thus “border shifts” in the future. The spectrum ranges from a time of absolute hope and the right not to know to the idea that the birth process can be completely controlled by scientific and technical methods. Both ideas also influence the extent of “self-determination” granted to women during childbirth. Another type of demarcation is the transition between physiological and pathological development. This will be increasingly discussed as obstetrics and midwifery evolve [38,39,40].

From an *action theory perspective*, health behaviour of pregnant women plays an important role in a milieu-dependent context, while symbolic interactionism deals with the question of how pregnancy/motherhood/non-pregnancy are symbolically charged and communicated [1,15,37,41,42,43]. Social practices of pregnancy and motherhood differ and depend on socio-legal conditions, cultural traditions and conventions and therefore also on the social milieu [44,45]. Since belonging to a social milieu contributes to the reproduction of social inequality, it also leads to health inequalities during pregnancy: Women from socially deprived backgrounds are less likely to take advantage of antenatal care [46] and are more likely to suffer from negative outcomes such as preeclampsia, premature birth or miscarriage [47,48]. They are more likely to suffer from pregnancy-related illnesses such as gestational diabetes [49] or hypertension [50]. They are more likely to engage in risky behaviour (e.g., nicotine abuse) [51] and are more often affected by psychosocial vulnerability [52,53]. The social context influences a variety of aspects related to pregnancy, including the gestational age [54], the corresponding probability of teenage pregnancies [55], the probability of being single at the time of the first pregnancy [56], the decision for or against an abortion in the case of unwanted pregnancies [57] and the mode of delivery in terms of the probability of a caesarean section [58,59,60]. The reasons why women want to become pregnant depend on the social norms that are reproduced in different social backgrounds. This diversity of norms results in a wide range of social expectations that act as a “social corset” on female individuals because they severely restrict their individual options for action. This social corset also influences the subjective experience of pregnancy and the milieu-dependent communication of pregnancy itself and pregnancy complaints in relation to socially constructed discourses of truth [1,37,44]. From an action theory perspective, it is also possible to resist imposed roles and expectations or renegotiate them, provided that there is active reflection on the fact that previous decisions were made on the basis of norms [4,5,6,61]. Foucault refers to this as the “will to disobey” [62], whereby social action is not possible without norms [4,5,6,61], meaning that in the case described, existing norms are replaced by others [62]. This can be seen in the context of pregnancy and childbirth, in the case when alternative birthing methods are being promoted. Alternative birth methods often involve a rejection or modification of the standard, medically dominated approach to childbirth. These methods, sometimes referred to as “natural birth” or “homebirth”, are influenced by diverse factors like a desire for greater autonomy, distrust of medical interventions, and beliefs in the body’s natural ability to birth [63]. The decision to choose non-traditional family constellations can also be analysed within this context [64]. Sociologically, pregnancy begins not with biological conception but with its social and institutional recognition. The foetus becomes part of the family system through pregnancy rituals, as with the infant after birth (depending on the milieu!). The decision for or against a child is also a social act and depends on the family’s value system and is therefore usually not based exclusively on the will of the pregnant woman [1,37,44]. For midwives, it can be noted that reproduction, pregnancy and childbirth are also social processes. This applies both to the decision for or against having children and to the reasons given and the way in which reproductive decisions are made and communicated.

## 5. Women’s Health and Social Construction of Gender Roles

Pregnancy and birth are social events [1,37,44] and are therefore also linked to gender-specific role expectations. Sociological theories postulate that individuals face each other as role bearers in the various social subsystems and fields and must constantly fulfil role expectations in order for society to function [65]. This means that women become pregnant in accordance with their gender roles because society expects them to do so or because they assume in certain contexts that this is what society expects of them. These role expectations are conveyed to girls and women during socialization, meaning that gender roles form the “social corset” described above. Society also has (re-)produced role expectations of the sexes, which influence gender-sensitive socialization from birth (although with significant differences in social milieus with regard to the value of individual underlying norms) and shape the social gender [66,67,68]. Underlying gender stereotypes shape the entire course of life [68,69,70] and influence health care in a variety of ways, as milieu-specific role expectations for the sexes also influence health and health behaviour of women and men [71]. For example, women are generally considered to be underserved compared to men in pain medication since gender influences the likelihood of receiving adequate pain care [71].

Sexual and contraceptive behaviour is also social behaviour that follows milieu-specific role expectations [57]. Pregnancy is highly relevant for all social systems and fields from both a micro- and macrosociological perspective, which is why sexual and reproductive behaviour has always been regulated [12,14,72,73,74]. Current debates about the legality of abortion (but also on the permissibility of contraception) show that decisions about a woman’s own physiology and organism are socially embedded and are not exclusively based on self-determined decision-making [75]. Against this background, aspects relating to the gender of individual women are always linked to social discourse and evaluations [72,73,74,76], as illustrated by the debate over what swimwear women should be allowed to wear [77,78].

These complex conditions must be taken into account when discussing aspects of self-determined pregnancy and birth. The conditions for exercising health rights are created in the social milieus, and discourses in the context of pregnancy, birth and the postpartum period follow role expectations and socially constructed norms and moral concepts [1,33,37,44,79,80]. For midwifery practice, the conclusion is that women’s health goes far beyond the discipline of gynaecology because women’s health is centrally influenced by gender-specific role expectations that exist for women.

## 6. Breastfeeding as a Social Process

Breastfeeding behaviour and the decision for or against breastfeeding are also influenced by a variety of social factors and should be considered in the context of a person’s life situation and sociodemographic factors [81,82]. Risk factors for insufficient breastfeeding include low maternal age, premature birth, nicotine abuse, as well as siblings who need to be looked after by their mother at the same time [83]. The most important influencing factor is education [84,85]. In Germany, the probability of a child being breastfed is 5.77 times higher for women with the highest level of education compared to women with the lowest educational level [83]. The regression model indicates that the likelihood of breastfeeding is not directly dependent on health literacy [86], but influencing factors related to education are to be expected here. For example, the proportion of people with low health literacy is many times higher among those with a low level of education. In most studies, “level of education” is generally classified as the highest school-leaving qualification, while “health literacy” is determined using questionnaires. The term refers to a person’s ability to find, understand, evaluate and apply health information in order to make informed decisions about their own health [84,85,86]. In general, health literacy is not in good shape: Approximately 50% of women of reproductive age in Germany have “fairly low” or “low” health literacy, meaning that they are unable to understand even simple health-related information [87]. This includes health-related information provided by midwives and information on breastfeeding. Training for fathers has also been shown to increase the likelihood of women breastfeeding their children, as study results from Iran suggest [88].

Many of the factors that influence the decision to breastfeed arise from the social environment (including workplace organization, level of education, shame or institutional barriers to breastfeeding in public [89]). A Belgian study found that one of the main reasons for the early cessation of breastfeeding is the need for the mother to return to work within a short period of time [90]. This suggests that the decision for or against breastfeeding is rarely made by the mother alone but is mediated in complex negotiation processes between the mother and the social systems surrounding her. The duration of breastfeeding is therefore not the sole responsibility of the mother but must be located within the framework of collective social responsibility. In many social contexts, there is still no supportive environment that enables breastfeeding [91]. Although the Maternity Protection Act in Germany, as an example, very explicitly stipulates that breastfeeding mothers must be released from work by their employer for at least two 30 min periods or one one-hour period per day for breastfeeding until their child’s first birthday if they so desire [92], Germany is considered only partially breastfeeding-friendly [93], as are other Western economies [94,95]. A recent study commissioned by the German Federation of Trade Unions points to significant deficits in the implementation of a breastfeeding-friendly environment, which is why many women who were employed while breastfeeding stated that they only breastfed outside working hours. When asked about their motives, 41% stated that they had voluntarily refrained from breastfeeding at work, presumably as a result of the conditions at work and/or the working atmosphere, which were experienced as unsuitable for undisturbed and private breastfeeding at work. Twelve per cent stated that they were expected to breastfeed only outside working hours [96]. Similar deficits are also evident in many other Western countries [91,97,98], and support from employers and the workplace environment is often perceived as inadequate [99]. So far, there are no studies on whether special workplace programs help to increase the duration of breastfeeding, according to the conclusions of a Cochrane Review [100].

There is a correlation between the termination of breastfeeding and the resumption of employment, which highlights the social aspects involved and must therefore also be analysed in sociological discourse. It is generally assumed that we face each other as role players in all social subsystems and that we fulfil and reproduce role expectations through social action [65]. This also applies to the category of gender: stereotypes and gender-related role expectations shape not least the professional context [66,67,68,69,70] and also have a significant influence on health behaviour and health care, often disadvantageously for women, since the health system does not yet have a sufficiently gender-sensitive orientation [71]. This means that breastfeeding is also subject to gender-specific role expectations [101]. According to Michel Foucault’s poststructuralism, breastfeeding women are exposed to a system of self- and external monitoring (“panopticon”) in public [102,103], and this takes place in a social environment that is perceived as hostile to breastfeeding [104], which so far insufficiently recognizes breastfeeding as a sexual and reproductive health right [105].

Breastfeeding at the workplace can lead to confusion regarding a woman’s role (professional role in the company or maternal role), which is usually associated with a lack of competence or is even considered a taboo within social systems [96,97,98,99,100,106].

The sexual disposition, a term used to describe the way in which society views and regulates sexuality, also contributes to a lack of willingness to breastfeed [12,107,108]. Social and media discourses mainly address the sexual nature of the female breast [81,103,109], which results in increased problems of natural maternal body perception [81,109,110] so that younger women are more likely to refrain from breastfeeding for aesthetic reasons [81,111]. The sexual connotation also results in both mothers and the social environment perceiving breastfeeding in public as unpleasant [81]. As a sexual object, the female breast is thus exposed to social norms and regulations (e.g., regarding its coverage), which admittedly differ depending on the cultural background [77,78,81,112]. In the 21st century, the fragmentation of social structures and norms means that different discourses increasingly have to be addressed in conjunction: breastfeeding touches on the sexual disposition as well as discussions about the functional emancipation of women, since breastfeeding is also associated with the reduction and exposure of the mother’s body for the benefit of the child’s well-being [81,101,112,113,114]. These findings highlight that midwives must recognize how social pressures and norms may shape a woman’s choices about breastfeeding.

## 7. Implications for Midwives

Midwives play a particularly important role in accompanying clients, especially in the reproductive phase, which means that they touch on many social levels: *macrosociologically*, because by providing care, they satisfy the social expectation that pregnant women have of consulting experts, for which they have been qualified and legitimized within an institutionalized training process [1,3,5,6,7,8,9]. At the *meso-level*, midwives act as a link between the individual needs of their clients and societal expectations of pregnancy care by working with other health care providers to ensure comprehensive care [33,44,45,115,116,117]. From a *microsociological* perspective, they should act as health advocates for their clients [118], although they will encounter a wide range of norms and behavioural patterns, as discourses on sexuality and reproduction are negotiated differently in different social milieus [1,37,44]. In pregnancy care, the standards of care must be measured against the expectations of the family’s social subsystem, which, as a microcosm, catalyses the specific ideas of pregnancy and birth of the social milieu to which it belongs [33,44,45,115,116,117]. Health promotion and empowerment can therefore only succeed if they follow the principles of targeting and tailoring [119,120,121]. Targeting refers to the needs/support requirements of the specific social milieu of the pregnant woman and takes into account the milieu-specific values and norms, including the autonomy granted to the pregnant woman in terms of decision-making [119,120,121]. Tailoring then addresses the needs and support requirements of an individual pregnant woman in the context of her social situation, considering her health literacy [119,120,121]. It should be noted that midwives also have role expectations in practicing their professional role, which differ in the various social milieus [122,123,124]. They also have formative power by bringing their own values and moral concepts into individual care situations, which can lead to a factual subordination of the client’s ideas to those of the midwife as a professional expert, despite the primacy of “self-determined pregnancy, birth and motherhood” [4,6,7,8,9,10,11,125,126]. Midwives should be self-critically aware of this position, especially in the context of the further development of gender roles and family models [127,128].

From a sociological perspective, midwifery care for women’s health is not only about empowering girls and women and their children; it is also about understanding the social dimensions of health, such as the reproduction of social inequality through social norms in the context of existing concepts of health and illness. It also means taking into account social determinants of health, including the influence of the social environment, family structures, and the socio-economic conditions of their clients in their own professional actions [67].

Acting sociologically as a midwife means that in every care situation, it must be taken into account that clients have a milieu-specific, culturally influenced value system that may differ from that of the midwife. The client’s value system influences her (reproductive) behaviour. Midwives must internalize that every social action of their clients is norm-related, as is every action of the midwife. Reflecting on this helps midwives to avoid condemning clients for behaviour that is harmful to their health (“don’t blame the victim!”) and imposing their own values in order to promote equality and inclusion in reproductive health care [119,120,121,122,123,124,125,126,127,128]. Against this background, there is a need to develop care models that emphasize the diversity of values [129]. Value pluralism, i.e., the recognition of different values, is an important aspect of midwives’ work, as they care for women in very different life situations and with diverse wishes and needs. Midwives must be able to reflect on their own values while respecting the values of the women they support. This requires a high level of ethical competence and a willingness to engage with different perspectives [130]. It can be helpful to conduct a detailed social anamnesis at the beginning of care and to use a questionnaire to identify the values specific to the patient’s social environment [131].

## 8. Limitations

This paper was designed as a narrative review, which imposes limitations that must be taken into account when evaluating the aspects presented in the paper. Specifically, it cannot be ruled out that there may be a selection bias, as the studies included were researched in PubMed, Google Scholar, and relevant journals but did not meet the strict criteria of a systematic search, such as those used in systematic reviews. For example, the suitability of studies was not assessed in terms of methodological characteristics but solely on the basis of thematic relevance, which may also result in a confirmation bias. Publication bias cannot be ruled out either, as only published literature was included without sufficient reflection (or the possibility of reflection) on whether there could be contrary results that contradict the theses presented here. There were no inclusion or exclusion criteria regarding study design. The central function of the paper was not to evaluate the state of research or the evidence of existing research results, but rather to provide midwives with an overview of sociological aspects of pregnancy and birth as a narrative review, because previous overview articles are lacking despite the high relevance of the topic. Accordingly, a lack of critical appraisal can be assumed, characterized by weak evidence remaining unquestioned and unweighted alongside robust findings. Another key limitation that must be pointed out is that (also for linguistic reasons) the study primarily took into account studies from Central Europe and North America. This implicitly adopts an Anglo-European perspective, leaving it unclear whether social aspects and cultural practices surrounding pregnancy and childbirth in other cultures (e.g., sub-Saharan Africa, the Middle East, and the Far East) have been adequately considered. Future research could explore how tailored midwifery interventions succeed across different socioeconomic or cultural groups.

## 9. Conclusions

Pregnancy and birth are social processes based on norms and role attributions: in many respects, “decisions” about one’s own ability to reproduce are only partially “self-determined”. They are not independent of rules in the context of social norms and milieu-specific role expectations. The promotion of women’s health depends on how milieu-specific norms and logics of action are understood. This implies the necessity for all obstetric professions to critically examine aspects of the sociology of health [132] in order to provide women and their families with appropriate, evidence-based and client-centred care in the context of pregnancy, birth and the postpartum period against the background of constant social change [1,3,133]. Proposals for behavioural change, regardless of the health profession that formulates them, can only be heard if they are not removed from the social context. Therefore, basic knowledge of sociological aspects of pregnancy, birth and breastfeeding must be taught in the basic studies of midwifery [134]. This means that sociology should be established as a core module in midwifery bachelor’s degree programs in order to teach students about the importance of the social environment as a central factor influencing health-related decisions in the context of pregnancy and childbirth [134]. In Germany, in-depth sociological knowledge has even been incorporated into the new Midwifery Act in 2020: graduates should be enabled to promote “the autonomy and self-determination of women, taking into account their rights, their specific life situation, and their ethnic, social, biographical, cultural, and religious background” [135]. In addition to thematic integration in specific modules in the bachelor’s program [134,135], further in-depth study can also be undertaken in master’s programs or as part of continuing education in advanced midwifery practice [136].

The implications for midwives can be summarized as follows:

1. Midwives work in a variety of social systems (including hospitals, outpatient clinics, families and social services) and must fulfil different social role expectations in each case.
2. Midwives work much more closely within the social system of the family than other professional groups (e.g., doctors or social workers). Accordingly, in-depth knowledge of family sociological aspects is necessary, as these have a central influence on the actions of family members and thus also on the client.
3. Diverse values and milieu-specific social gender stereotypes have a central influence on women’s reproductive and health-related decisions.
4. Midwives also come from different milieus and accordingly have a heterogeneous value system. This should not lead midwives to judge clients based on their values or impose their own values on them.
5. The central function of midwives is to promote women’s self-determination in the context of pregnancy and birth. This can only be achieved if midwives identify their clients’ value systems and make health-promoting suggestions within this context.
6. Successful health promotion in the context of pregnancy and birth can only be achieved if the care model is based on the criteria of tailoring and targeting.

## Data Availability

Not applicable.
